# TaME-seq2: tagmentation-assisted multiplex PCR enrichment sequencing for viral genomic profiling

**DOI:** 10.1186/s12985-023-02002-5

**Published:** 2023-03-08

**Authors:** Alexander Hesselberg Løvestad, Milan S. Stosic, Jean-Marc Costanzi, Irene Kraus Christiansen, Hege Vangstein Aamot, Ole Herman Ambur, Trine B. Rounge

**Affiliations:** 1 Faculty of Health Sciences, OsloMet - Oslo Metropolitan University, Oslo, Norway; 2 Department of Microbiology and Infection Control, Akershus University Hospital, Lørenskog, Norway; 3 Division of Medicine, Department of Clinical Molecular Biology (EpiGen), Akershus University Hospital and University of Oslo, Lørenskog, Norway; 4 Department of Research, Cancer Registry of Norway, Oslo, Norway; 5 Department of Pharmacy, Centre for Bioinformatics, University of Oslo, Oslo, Norway

**Keywords:** Intra-host variation, Library preparation, NGS, HPV, SARS-CoV-2, Virology, Genomics, Viral integration

## Abstract

**Background:**

Previously developed TaME-seq method for deep sequencing of HPV, allowed simultaneous identification of the human papillomavirus (HPV) DNA consensus sequence, low-frequency variable sites, and chromosomal integration events. The method has been successfully validated and applied to the study of five carcinogenic high-risk (HR) HPV types (HPV16, 18, 31, 33, and 45). Here, we present TaME-seq2 with an updated laboratory workflow and bioinformatics pipeline. The HR-HPV type repertoire was expanded with HPV51, 52, and 59. As a proof-of-concept, TaME-seq2 was applied on SARS-CoV-2 positive samples showing the method’s flexibility to a broader range of viruses, both DNA and RNA.

**Results:**

Compared to TaME-seq version 1, the bioinformatics pipeline of TaME-seq2 is approximately 40× faster. In total, 23 HPV-positive samples and seven SARS-CoV-2 clinical samples passed the threshold of 300× mean depth and were submitted to further analysis. The mean number of variable sites per 1 kb was ~ 1.5× higher in SARS-CoV-2 than in HPV-positive samples. Reproducibility and repeatability of the method were tested on a subset of samples. A viral integration breakpoint followed by a partial genomic deletion was found in within-run replicates of HPV59-positive sample. Identified viral consensus sequence in two separate runs was > 99.9% identical between replicates, differing by a couple of nucleotides identified in only one of the replicates. Conversely, the number of identical minor nucleotide variants (MNVs) differed greatly between replicates, probably caused by PCR-introduced bias. The total number of detected MNVs, calculated gene variability and mutational signature analysis, were unaffected by the sequencing run.

**Conclusion:**

TaME-seq2 proved well suited for consensus sequence identification, and the detection of low-frequency viral genome variation and viral-chromosomal integrations. The repertoire of TaME-seq2 now encompasses seven HR-HPV types. Our goal is to further include all HR-HPV types in the TaME-seq2 repertoire. Moreover, with a minor modification of previously developed primers, the same method was successfully applied for the analysis of SARS-CoV-2 positive samples, implying the ease of adapting TaME-seq2 to other viruses.

**Supplementary Information:**

The online version contains supplementary material available at 10.1186/s12985-023-02002-5.

## Background

New sequencing methods have enabled deep diving into viral genomics and viral-host interactions. Nearly all cases of cervical cancer have persistent infections with human papillomavirus (HPV) as a causative agent [1]. Recent studies of HPV intra-host variation have revealed the presence of minor nucleotide variants (MNVs) below the consensus sequence level that can be of clinical relevance for the development of HPV-induced cervical cancer [2, 3]. Additionally, the clinical relevance of intra-host MNVs is not limited to HPV infections, as shown in previous deep-sequencing studies of other viral infections [4–7]. Furthermore, the integration of the HPV genome into the human genome is frequently observed [8] and is considered a driving event in HPV-induced cancer development [9].

**Fig. 1:**
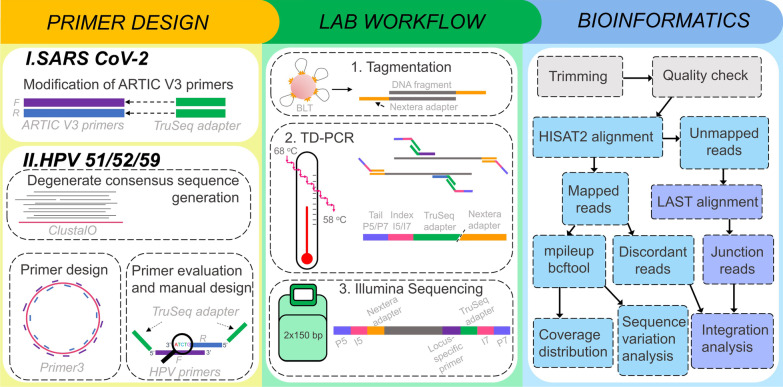
Overview of the TaME-seq2 workflow. The workflow is divided into three steps, primer design shown separately for SARS-CoV-2 and HPV51/52/59, lab workflow consisting of tagmentation step, touch-down PCR and Illumina sequencing, and bioinformatics analysis represented as a flowchart

A tagmentation-assisted multiplex PCR enrichment sequencing protocol, TaME-seq, for deep sequencing of HPV, has previously been developed [10]. This protocol allows simultaneous identification of the consensus sequence, low-frequency variable sites, and chromosomal integration events within clinical samples. Similar methods generally allow the analysis of either HPV genomic variability or integrations and are also less cost-efficient [11–13]. TaME-seq has been successfully applied to study high-risk (HR) HPV types, HPV16, 18, 31, 33, and 45 [10, 14, 15]. This study presents TaME-seq2 with an updated laboratory workflow and bioinformatics pipeline (Fig. 1). In addition, the HPV repertoire is expanded to include three HR-HPV types, HPV51, 52, and 59. Investigating intra-host genomic events in less studied HR-HPV types broadens our understanding of mechanisms behind HPV-induced carcinogenesis, while also giving insight into why some HPV types have a higher carcinogenic potential than others. Therefore, our long-term goal is to include all HR-HPV types in the TaME-seq2 repertoire. Furthermore, we present a proof-of-concept that TaME-seq2 can be used for SARS-CoV-2 sequencing and easily be applied to both DNA and RNA viruses.

## Results

Summarized sequencing results for each virus type are shown in Table 1. Detailed sequencing results of 36 cervical cell samples positive for HPV51/52/59 (12 per type), HPV harbouring plasmid samples with targeted types (two per type), 23 SARS-CoV-2 positive samples, and negative controls are presented in Additional file 1: Table S2. The total number of generated raw reads from all HPV samples was 553.3 million, of which 72.9 million mapped to HPV. The percentage of raw reads mapping to the target HPV was 14.3% for all samples, and after the low-quality samples were filtered out (three HPV51, five HPV52, and five HPV59), the percentage increased to 15.5%, while these values were 33,5% and 38.1% for the trimmed reads, respectively. Only 0.4% of the HPV reads mapped to off-target HPV types. No significant difference in the number of off-target mapping reads was found between samples with single and multiple HPV infections (Wilcoxson test, *p* = 0.15). Mean sequencing depth ranged between 0.06× and 82 704×. In total, 23 HPV-positive samples, excluding positive plasmid controls, passed the threshold of 300× mean depth and were submitted for further analysis.

**Table 1:** Overview of the sequencing results of HPV 51/52/59 positive cervical-cell samples and SARS-CoV-2 positive samples

Virus type	# of samples	Mean # of raw reads (mil)	Mean # of trimmed reads (mil)	Mean # of reads mapped to target (mil)	Mean % raw reads mapped to target (%)	Mean % trimmed reads mapped to target (%)	Mean coverage per sample	Mean % of genome covered by minimum		Variable sites in samples passing 300× mean coverage			
								10x (%)	100x (%)	Mean	Min	Max	Mean # of variable sites per 1 kb of genome
HPV51	12	22.7	9.0	2.7	15.5	37.3	24,911	89.4	83.4	21	4	53	2.7
HPV52	12	10.4	5.4	1.3	8.5	18.1	13,003	75.9	63.7	19	3	38	2.4
HPV59	12	8.6	3.4	2.0	16.2	38.2	14,893	74.6	57.9	20	5	30	2.5
SARS-CoV-2	23	23.0	18.2	1.3	5.4	12.5	3454	43.3	28.7	116	24	215	3.9

**Fig. 2:**
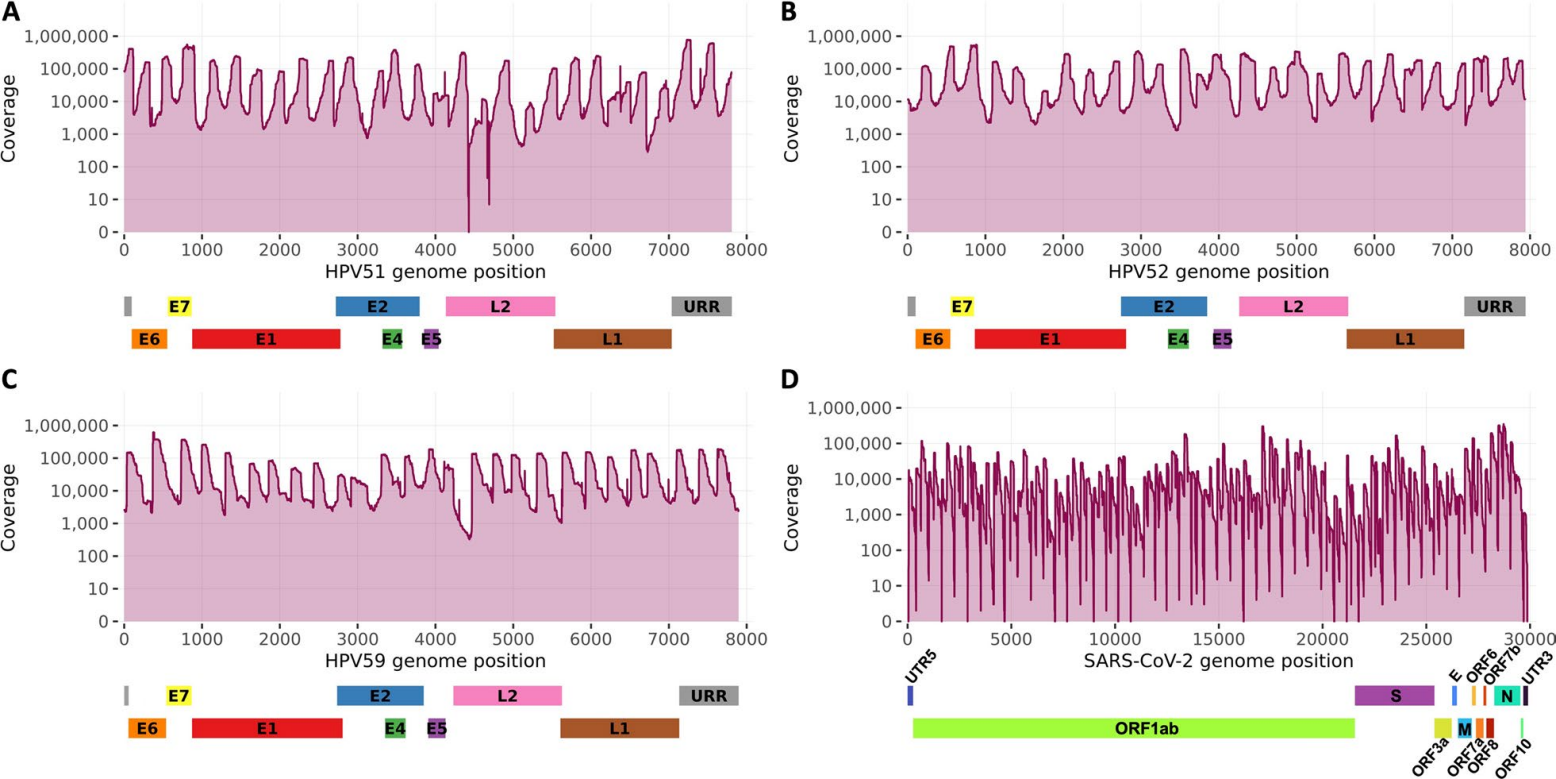
Sequencing coverage in representative HPV51, 52, 59, and SARS-CoV-2 positive samples. The coverage plots of **A** HPV51, **B** HPV52, **C** HPV59, and **D** SARS-CoV-2 aligned to the respective target HPV and SARS-CoV-2 genomes

Sequencing of SARS-CoV-2 positive samples resulted in 529.5 million raw reads, of which 28.8 million mapped to the SARS-CoV-2 genome. Seven samples passed the minimum requirement for subsequent analysis, with a mean percentage of raw and trimmed reads mapping to SARS-CoV-2 being 17.3% and 39.9% respectively (Additional file 1: Table S2). Coverage plots of three representative samples from each HPV type and one SARS-CoV-2 positive sample are shown in Fig. 2.

### Analysis of viral deletion and viral integration into the human genome

Integration was found in only one within-run replicate 10a-HPV59/10b-HPV59. Two reported integration breakpoints were found in the HPV genes E2 and E5, and in the human chromosome 2 (Additional file 1: Table S3 and Figure S2). Deletions were found between 2348 and 3039 bp in 10a-HPV59, and between 2349 and 3036 bp in 10b-HPV59 corresponding to the segments of E1 and E2 genes (Additional file 1: Figure S1). In SARS-CoV-2 positive samples, neither viral deletion nor integration events were detected.

Moreover, two short regions with a coverage drop were observed in all HPV51 samples. The affected nucleotides positions were found between 4416–4430 and 4690–4692, corresponding to the L2 gene region, most likely due to suboptimal primer hybridization and/or poor alignment against the reference genome (Fig. 2A).

### Minor nucleotide variation and mutational signature analysis

**Fig. 3:**
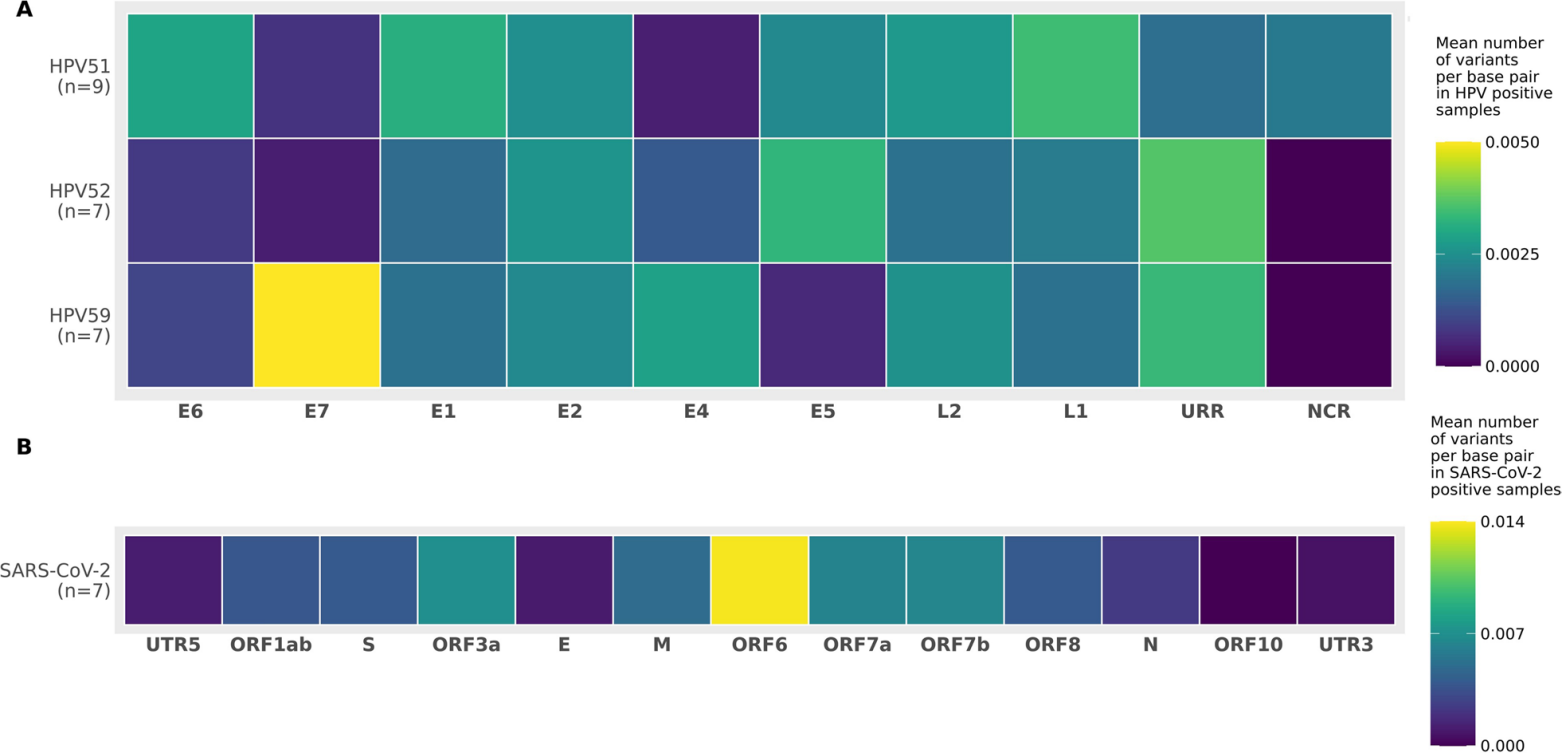
Mean number of variants per gene normalized by gene length presented as heatmap. **A** HPV51, HPV52, and HPV59 positives samples **B** SARS-CoV-2 positive samples

Minor nucleotide variation analysis identified 21, 19, and 20 average MNVs per sample in HPV51, 52, and 59 positive samples, respectively. The number of MNVs found within individual samples ranged from 3 to 53 irrespective of HPV type (Table 1, Additional file 1: Figure S3A). Moreover, MNVs’ positions were scattered throughout the genomes, with HPV59 E7 showing the highest degree of variation per base pair (Fig. 3A).

The mean number of variable sites in SARS-CoV-2 was 116 (Table 1, Additional file 1: Figure S3B). Accounting for gene length, ORF6 had the highest number of MNVs per base pair (Fig. 3B). The mean number of variable sites per 1 kb in SARS-CoV-2 was ~ 1.5× higher compared to HPV types (Table 1).

**Fig. 4:**
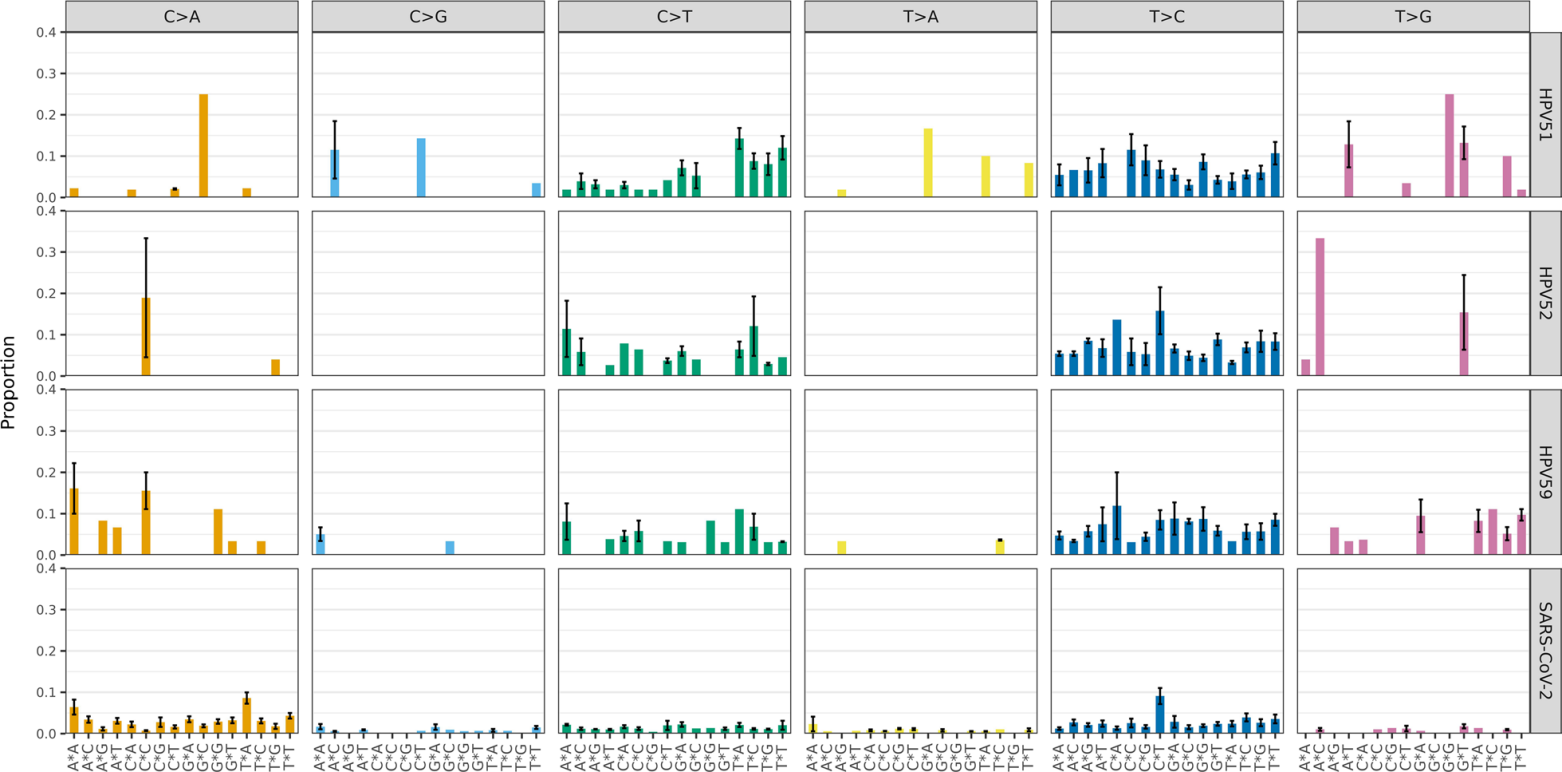
Mutational signatures in HPV51, HPV52, HPV59, and SARS-CoV-2 positive clinical samples. The mean proportion of 96 trinucleotide context types is shown below the plots across the different substitution categories. Error bars represent the standard error of the mean

Mutational signatures found in HPV51, 52, and 59 were almost identical, with C > T and T > C being the most prevalent substitutions regardless of the trinucleotide context and HPV type (Fig. 4). Out of all C > T substitutions detected, the ones in the trinucleotide context TCW (W is either A or T) were the predominant C > T substitutions in HPV51 samples. TCW trinucleotide context has previously been described as a target sequence of APOBEC3 proteins [16]. T > C and C > A substitutions were the most prevalent in SARS-CoV-2 samples (Fig. 4).

### Reproducibility of consensus sequences, MNVs, and mutational signatures between replicates

Technical between-run replicates of six HPV51 and nine HPV52 positive samples were used to assess the reproducibility of both consensus sequence identification and minor nucleotide calling. Replicates were tagmented, PCR amplified, and sequenced independently. In total, seven replicates did not meet the minimum requirements to be included in the analysis due to mean coverage being < 300× in one or both samples (n = 5) or having failed forward or reverse reactions in one of the samples (n = 2).

The sequencing results of the eight replicates included in the analysis are presented in Table 2. The results of two sequencing runs were significantly different (Wilcoxon paired two-tail test) in terms of the number of trimmed reads (*p* = 0.008), reads mapping to HPV (*p* = 0.008), reads mapping to the targeted HPV (*p* = 0.008), and mean coverage between pairs of samples (*p* = 0.008). However, the percentage of genome covered with > 100× and > 300× was unaffected by the sequencing run (Wilcoxon paired two-tail test, *p* = 0.2, and *p* = 0.5, respectively).

**Table 2:** Overview of the results of two sequencing runs of eight HPV-positive replicated samples

Sample ID	# of trimmed reads (mil)		# of reads mapped to HPV (mil)		# of reads mapped to target (mil)		Mean coverage		% Genome covered by minimum 100x		% Genome covered by minimum 300x	
	Run1	Run2	Run1	Run2	Run1	Run2	Run1	Run2	Run1 (%)	Run2 (%)	Run1 (%)	Run2 (%)
1-HPV51	10.8	4.5	2.8	0.8	2.8	0.8	27,662	8865	100	100	97	99
2-HPV52	5.1	2.2	0.5	0.2	0.5	0.2	5444	2872	100	100	96	97
3-HPV52	6.3	3.5	4.7	2.6	4.7	2.6	50,735	29,829	100	100	100	100
4-HPV51	12.6	3.3	8.4	1.3	8.4	1.3	71,702	13,628	100	100	100	98
5-HPV52	4.9	0.9	0.6	0.1	0.6	0.1	5834	1373	100	100	95	84
6-HPV52	5.9	2.5	0.8	0.4	0.8	0.4	8500	4124	100	100	98	99
7-HPV51	10.0	2.5	5.4	0.8	5.4	0.8	52,417	8402	100	100	100	97
8-HPV52	9.5	4.5	8.8	3.0	8.8	3.0	82,704	32,611	100	100	100	100

Consensus sequences between replicates were identical, except for a few positions, ranging between 1 and 6 nucleotides (Table 3). Conversely, replicates exhibited a large difference in MNVs between the two runs (Table 4). Identical MNVs shared between replicates ranged between zero and three. However, the total number of MNVs in replicates detected between the two runs was not significantly different (Wilcoxon paired two-tail test, *p* = 0.3).

**Table 3:** Overview of major nucleotide calling results used to generate consensus genome sequences in eight HPV replicates

Sample ID	# of identically called major nucleotides in Run1 and Run2	# of major nucleotides called only in		# of major nucleotides called differently between runs	Mean coverage of the major nucleotide	
		Run1	Run2		Run1	Run2
1-HPV51	7766	1	6	0	27,711	8886
2-HPV52	7941	0	0	0	5429	2869
3-HPV52	7942	0	0	0	50,585	29,773
4-HPV51	7788	1	6	0	71,638	13,617
5-HPV52	7941	1	0	0	5816	1372
6-HPV52	7942	0	0	0	8474	4117
7-HPV51	7787	3	2	0	52,371	8405
8-HPV52	7942	0	0	0	82,431	32,528

For each pair of samples, the number of identical major nucleotides between runs, the number of nucleotides called in one of the runs, and the number of called nucleotides differing between runs are shown. The mean coverage of the called major nucleotides in two runs is also presented

**Table 4:** Overview of MNV calling results in eight HPV replicates

Sample ID	# of identically called MNVs in Run 1 and Run 2	# of MNVs called only in	# of MNVs called differently between runs	Total # of MNVs found		Mean coverage of the called MNVs	
		Run1	Run2	Run1	Run2	Run1	Run2
1-HPV51	1	12	11	13	12	37	219
2-HPV52	0	22	22	22	22	60	32
3-HPV52	1	2	7	3	8	653	77
4-HPV51	2	5	7	7	9	465	100
5-HPV52	1	24	12	25	13	34	41
6-HPV52	1	11	29	12	30	39	53
7-HPV51	0	4	5	4	5	1231	23
8-HPV52	3	0	5	3	8	676	282

For each pair of samples, the number of identical MNVs between runs, the number of MNVs called only in one of the runs, the number of MNVs differing between runs is shown, and the total number of MNVs found. Mean coverage of the called MNVs in two runs is also presented

To further investigate whether the observed difference would affect the calculation of gene variability, MNVs in different runs were grouped by the genes they occurred in, regardless of the HPV type, and counted (Additional file 1: Table S4). Even though MNVs were at different positions, their numbers per gene were not significantly different between runs (Wilcoxon paired two-tailed test, *p* = 0.4).

Furthermore, the reproducibility of mutational signature analysis was also assessed. In both sequencing runs, regardless of the HPV type, the same pattern of C > T and T > C substitutions was detected (Additional file 1: Figure S4). The number of all observed substitutions in their respective trinucleotide context was not significantly different between runs, as well as their mean proportions (Wilcoxon paired two-tailed test, *p* = 0.4).

## Discussion

TaME-seq2 enables in-depth analysis of DNA and RNA viruses by exploiting the latest kits and efficient software. Compared to the previous version of TaME-seq, the bioinformatics pipeline is approximately 40× faster without affecting the analysis quality. The laboratory workflow was improved by implementing the latest Nextera tagmentation kit accompanied by touchdown PCR, known to increase the amplification yield and decrease off-target amplification [17]. Adding unique dual indexes minimizes the risk of calling erroneous low-frequency variants due to index hopping.

The number of HPV samples passing the threshold is affected by lower initial DNA input and/or low viral load in samples, a general feature of multiplex target enrichment protocols and reported for the previous version of the protocol [15]. In SARS-CoV-2 samples, the high number of Illumina-tail-extended primers was most likely responsible for the primer-dimer formation, in turn causing reduced amplification yield. The sequencing depth of SARS-CoV-2 samples was in some positions very low (Fig. 2D), probably caused by the primer pools not being designed for the TaME-seq2 protocol but for tiling amplicon sequencing, affecting amplification performance. Even though the method was successfully applied as a proof-of-concept, further optimization of the laboratory workflow and primer pools is recommended.

The only deletion and viral integration into the human genome were found in the HPV59 positive within-run replicate, indicating a repeatability in detecting deletion/integration events as shown for the previous iteration of the protocol [10]. Detected breakpoints in early genes accompanied by complete or partial deletion of E1 and E2 have frequently been observed for HPV integrations [12]. Integrations into the human genome were not found in SARS-CoV-2 positive samples, confirming previous findings [18].

The mean number of MNVs in included HPV types was similar and in line with previous studies [14, 19–22]. In SARS-CoV-2 samples, a higher mean number of detected MNVs compared to HPV-positive samples is probably the consequence of the significant difference in genome size between HPV and SARS-CoV-2. Furthermore, a higher mutation rate is expected in RNA viruses, which replicate using low-fidelity RNA-dependent RNA polymerase [23]. As the SARS-CoV-2 samples exhibited somewhat lower mean coverage and sequencing depth compared to HPV, it can be expected that higher sequencing coverage would increase the number of detected MNVs.

Mutational signatures found in HPV51, 52, and 59 were almost identical, with C > T and T > C being the most prevalent substitutions regardless of the trinucleotide context and HPV type. The same signatures were also found in previously assessed types HPV16, 18, 31, 33, and 45 [14, 19]. T > C and C > A substitutions were the most prevalent in SARS-CoV-2 samples, as shown in similar studies [6, 7].

The reproducibility of the TaME-seq2 performance was assessed with eight HPV-positive replicates, which underwent independent library preparation and sequencing. As the number of samples on the flow cells differed between the sequencing runs, the number of generated reads between runs differed. However, the overall performance of TaME-seq2 in terms of the percentage of genome covered with > 300× per sample replicate was not significantly different between the two runs.

Consensus sequences were identical between the two runs irrespective of HPV types, except for a few low coverage positions in some of the samples. On the other hand, the shared number of identical MNVs between replicates of each pair was low. Identification of low-frequency variants depends mainly on the coverage at the given site. In our previous study, the number of detected low-frequency MNVs increased with the mean coverage, reaching a saturation plateau at ~ 12 000× [10]. As identical nucleotides in replicated samples might have different coverage, the number of detected MNVs at identical positions is expected to differ. Moreover, only a small proportion of HPV genomes in the sample harbours MNVs. As tagmentation, PCR amplification, and sequencing are stochastic processes favouring higher-frequency variants within samples, many low-frequency variants can be expected to stay undetected. Especially PCR amplification introduces biases, favouring amplification of higher-frequency variants [24], thereby altering the analysed intra-host variation composition in a stochastic manner. Even if the sequencing depth of the whole HPV genome is above the saturation plateau, it cannot be expected that all identical MNVs would be detected after the sample replicates are tagmented, PCR-amplified, and sequenced independently.

The total number of detected MNVs between replicates sequenced in two different runs was not significantly different. Regardless of HPV type, the gene variability did not differ significantly between independent runs. Furthermore, assigning the MNVs by their substitution type in a trinucleotide context did not show a significant difference between runs or HPV types (Additional file 1: Figure S4). Overall, the results indicate that the method is suitable for investigating intra-host MNV diversity and is in line with similar studies [2, 3]. The mutational signature analysis is robustly detecting the specific within-patient mutation patterns.

## Conclusion

TaME-seq2 proved well suited for both consensus sequence identification, low-frequency viral genome variation, and viral-chromosomal integration analysis. Now, the repertoire of TaME-seq2 encompasses HPV51, 52, and 59 in addition to HPV16, 18, 31, 33, and 45. Moreover, with the addition of Illumina TruSeq-compatible adapter tails to previously developed primers, the same method was successfully applied as a proof-of-concept for the analysis of SARS-CoV-2, implying the ease of adapting TaME-seq2 to a broader variety of viruses. However, further optimization of the laboratory workflow and primer pools is recommended.

## Methods

### Sample material

The study included the following material: (i) Cervical cell samples positive for HPV51, 52, and 59 (Additional file 1: Table S1). The samples are part of a research biobank and were collected between 2005 and 2008 from women participating in the cervical cancer screening program in Norway [25, 26]. These samples had either a single infection or multiple infections with at least one of the targeted types (n = 36, 12 per type). (ii) Paired technical HPV-positive replicates (n = 16), of which 15 pairs were sequenced in separate sequencing runs and one replicate pair sequenced within the same sequencing run. (iii) Positive controls, two HPV harbouring plasmid samples per targeted type from the Equalis global HPV DNA typing proficiency study 2019 [27] containing either a single HPV type or multiple HPV types. (iv) 23 SARS-CoV-2 cDNA samples from a study on suspected intra-hospital SARS-CoV-2 transmissions during the first wave of the COVID-19 pandemic [28]. Briefly, samples underwent reverse transcription in 10 μl reactions, containing 2 μl LunaScript^®^ RT SuperMix (NEB, Ipswich, MA) and 8 μl sample RNA with the following incubation program: 25 °C for 2 min; 55 °C for 10 min; 95 °C for 1 min.

### Laboratory workflow

A detailed workflow of the protocol can be accessed at: dx.doi.org/10.17504/protocols.io.dm6gpjxy5gzp/v1. The updated TaME-seq2 protocol includes a previously described protocol used for designing HPV51, 52, and 59 specific primers [10] (Fig. 1). SARS-CoV-2 primers were designed by adding Illumina TruSeq-compatible adapter tails to the 5’ends of the ARTIC Version 3 primer set designed by the Artic Network [29].

TaME-seq2 compatible unique dual indexes were designed using the IDT^®^ for Illumina^®^ DNA/RNA UD Indexes as a template and adding Nextera-compatible adapter tails as described previously [10, 30]. All primers were synthesised by Thermo Fisher Scientific, Inc. (Waltham, MA).

HPV and SARS-CoV-2 specific forward and reverse primer pools were prepared separately in equal volumes and diluted to a concentration of 15 µM. The unique dual indexes were diluted to 10 µM and corresponding i5/i7 pairs were combined.

The updated TaME-seq2 protocol includes tagmentation and post-tagmentation clean-up using the Illumina^®^ DNA Prep Tagmentation kit (Illumina, Inc., San Diego, CA) according to the manufacturer’s recommendations but using half the recommended reaction volumes. Qiagen Multiplex PCR Master Mix (Qiagen, Hilden, Germany) was prepared and mixed with tagmented DNA still attached to the bead-linked transposomes. DNA-containing master mixes were divided into two separate PCR reactions of equal volumes, and forward or reverse virus-specific primer pools were added to the respective PCR reactions. Primer pool concentrations were 0.6 µM and the final concentration of i5/i7 index primers was 0.8 µM per 25 µl PCR reaction.

The updated protocol includes a touchdown PCR consisting of 5 min initial denaturation and hot start at 95 °C; 10 touchdown cycles consisting of denaturation at 95 °C for 30 s, annealing at 68 °C for 90 s, decreasing by 1 °C per cycle, and elongation at 72 °C for 30 s; 26 cycles with the fixed annealing temperature at 58 °C for 90 s with the PCR cycling parameters as in the previous cycling step; and final extension at 68 °C for 10 min. The bead-linked transposomes were removed before the forward and reverse reactions were pooled in equal volumes and submitted to clean-up and two-sided size selection using purification beads (Illumina^®^ DNA Prep Tagmentation kit) according to the manufacturer’s instructions. The libraries were then washed three times using a 0.65 × ratio of Sample Purification beads (Beckman Coulter, Brea, CA) to remove excess primer-dimers and short fragments < 300 bp.

Due to the detection of primer-dimer excess (⁓150 bp) in the SARS-CoV-2 sample libraries after size selection and clean-up, a DNA gel-extraction was performed using the Wizard^®^ SV Gel and PCR Clean-Up System (Promega, Madison, WI) to extract fragments > 300 bps.

Quality and quantity of pooled libraries were assessed on Agilent 2100 Bioanalyzer using Agilent High Sensitivity DNA Kit (Agilent Technologies Inc., Santa Clara, CA) before sequencing on the NovaSeq 6000 platform with the SP Reagent Kit v1.5 (Illumina, Inc., San Diego, CA). Samples were sequenced as 151 bp paired-end reads.

### Bioinformatic workflow

The TaME-seq2 bioinformatic pipeline includes trimming of raw pair-end reads by removal of adapters, virus-specific primers, and nucleotides with a quality < 20 by cutadapt (v3.4) and quality check by FastQC (v.0.11.9) and MultiQC (v1.10.1). The trimmed reads were mapped to the virus-specific and human (hg38) reference genomes using HISAT2 [31] (v2.2.1). All available HPV reference genomes retrieved from the Papillomavirus Episteme (PaVE) [32] database were used in the reference genome file for HPV-positive samples, while NC_045512.2 was used for SARS-CoV-2 positive samples. Subsequently, mpileup from bcftool (v1.12) compiled the mapping statistics at a single nucleotide resolution. Mapping statistics and sequencing coverage from forward and reverse reactions for each sample were combined and visualised with an in-house R (v3.5.1) script, enabling evaluation of the method performance and downstream chromosomal integration and sequence variation analysis. This study excluded samples with < 300× mean sequencing depth from downstream analyses. This threshold may vary depending on the research aim.

### Integration analysis

Viral chromosomal integration analysis was performed as described previously [10]. In brief, discordant read-pairs from the HISAT2 mapping were identified, and potential integration sites were reported if ≥ 2 human-mapping reads exhibited unique start and/or end coordinates. The LAST aligner remapped HISAT2-unmapped reads to the reference genomes, thereby identifying junction reads. Positions covered with ≥ 3 junction reads, with unique start and end coordinates, were designated potential integration breakpoints. All viral-human integration points were validated by visualizing junction and discordant reads mapped to the identified human genome regions by Integrative Genomics Viewer (IGV, v2.5.3). Integration sites reported exclusively by read mapping to repetitive regions, split reads, and reads falsely identified as unique due to the few missing bases probably removed during trimming, were discarded.

### Minor nucleotide variant calling analysis

Variant calling was conducted using an in-house R script similarly as previously described [10]. Samples with failed forward or reverse reactions were excluded from MNV analysis. Nucleotides with a mean Phred score ≤ 30, nucleotides observed ≤ 3 in each position, and nucleotide positions with < 100× coverage were omitted from the analysis. All nucleotide variants for each position were counted from forward and reverse reactions separately. The most frequent variant in a position was designated Major while the second most frequent variant was designated MNV. If identified MNVs differed between forward and reverse reactions, the variant in the reaction with the highest coverage was used. A filtering step discarded MNVs with a frequency ≤ 1% within a sample. Detected MNVs of targeted HPV types found in the non-coding region (NCR) were manually investigated, discarding those present in homopolymeric regions (nucleotide repeated ≥ 5 times). Called MNVs were used in the mutational signature analysis classifying MNVs as either C > A, C > G, C > T, T > A, T > C, or T > G substitutions and further into 96 trinucleotide contexts and the proportional number of each substitution in each trinucleotide context per sample was calculated.

### Reproducibility and repeatability analysis

The reproducibility and repeatability of the method were assessed using within-run and/or between-run replicates. After removing samples that failed to meet the filtering criteria, eight between-run and one within-run pairs underwent variant calling and integration analysis. Consensus sequences were generated by combining the forward and reverse reactions and calling the nucleotide with the highest coverage in each position (min. 20×). The consensus sequences and MNVs identified between technical replicate pairs were then compared against each other.

## Supplementary Information

**Additional file 1**.** Table S1**: Overview of cytology-based and/or histology classification of clinical samples positive for HPV51, 52, and 59. (LSIL – low-grade intraepithelial lesion, ASC-US - atypical squamous cells of undetermined significance, HSIL- high-grade intraepithelial lesion, ASC-H - Atypical squamous cells- cannot exclude high-grade squamous intraepithelial lesion, CIN1- Cervical intraepithelial neoplasia grade 1, CIN2-Cervical intraepithelial neoplasia grade 2, ND-not determined, NR-not relevant).** Table S2**: Read counts and sequencing statistics of HPV positive cervical cell samples, HPV harboring plasmids (Equalis proficiency panel) and SARS-CoV-2 positive clinical samples (NA-not applicable).** Table S3**: Location of the integration breakpoints found in duplicates of 10-HPV59 sample and number of unique discordant read pairs and junction reads at the integration breakpoints.** Table S4**: Number of minor nucleotide variants (MNVs) grouped by HPV gene, identified in run 1 and run 2.** Figure S1**: HPV genome sequencing coverage in replicates 10a-HPV59 and 10b-HPV59.** Figure S2**. IGV visualization of HISAT2 and LAST alignments of 10a-HPV59 and 10b-HPV59 to human genome indicating HPV-human integration breakpoints. (a) Integration breakpoint between chromosome 2 (GRCh38/hg38) and HPV59 E2. (b) Integration breakpoint between chromosome 2 and HPV59 E5. HISAT2 reads are presented with dark gray color. Parts of the LAST reads mapping to human are light gray, while parts mapping to HPV59 are multi-colored. Red arrows point to the exact breakpoint positions.** Figure S3**. Number of minor nucleotide variants presented as violin plots across the different virus types, A) HPV52, HPV52, and HPV59; B) SARS-CoV-2. Violin plot shows the probable density of the data, using kernel density estimation. Box-and-whisker plots are added to show the median number (horizontal line), 25% and 75% percentiles (box), minimum and maximum values (whiskers).** Figure S4**: Mutational signatures found between technical replicates in the two sequencing runs. The mean proportion of 96 trinucleotide substitution types is shown below the plots across the different diagnostic categories. Error bars represent the standard error of the mean.

## Data Availability

The data presented in this article are not readily available because of the principles and conditions set out in the General Data Protection Regulation (GDPR), with additional national legal basis as per the Regulations on population-based health surveys and ethical approval from the Norwegian Regional Committee for Medical and Health Research Ethics (REC). Requests to access the data should be directed to the corresponding authors.

## Abbreviations

HPV: Human papillomavirus
SARS-CoV-2: Severe acute respiratory syndrome coronavirus 2
MNV: Minor nucleotide variant
TaMe-seq: Tagmentation-assisted multiplex PCR enrichment sequencing protocol
HR: High risk
